# Quantitative benefit–harm assessment for setting research priorities: the example of roflumilast for patients with COPD

**DOI:** 10.1186/s12916-015-0398-0

**Published:** 2015-07-02

**Authors:** Milo A. Puhan, Tsung Yu, Cynthia M. Boyd, Gerben ter Riet

**Affiliations:** Epidemiology, Biostatistics and Prevention Institute, University of Zurich, Zurich, Switzerland; Department of Epidemiology, Johns Hopkins Bloomberg School of Public Health, Baltimore, USA; Center on Aging and Health, Division of Geriatric Medicine and Gerontology, Johns Hopkins School of Medicine, Baltimore, USA; Academic Medical Center, Department of General Practice, University of Amsterdam, Amsterdam, Netherlands

**Keywords:** Benefit–harm assessment, Chronic obstructive pulmonary disease, Randomized trials, Research priorities

## Abstract

**Background:**

When faced with uncertainties about the effects of medical interventions regulatory agencies, guideline developers, clinicians, and researchers commonly ask for more research, and in particular for more randomized trials. The conduct of additional randomized trials is, however, sometimes not the most efficient way to reduce uncertainty. Instead, approaches such as value of information analysis or other approaches should be used to prioritize research that will most likely reduce uncertainty and inform decisions.

**Discussion:**

In situations where additional research for specific interventions needs to be prioritized, we propose the use of quantitative benefit–harm assessments that illustrate how the benefit–harm balance may change as a consequence of additional research. The example of roflumilast for patients with chronic obstructive pulmonary disease shows that additional research on patient preferences (e.g., how important are exacerbations relative to psychiatric harms?) or outcome risks (e.g., what is the incidence of psychiatric outcomes in patients with chronic obstructive pulmonary disease without treatment?) is sometimes more valuable than additional randomized trials.

**Summary:**

We propose that quantitative benefit–harm assessments have the potential to explore the impact of additional research and to identify research priorities Our approach may be seen as another type of value of information analysis and as a useful approach to stimulate specific new research that has the potential to change current estimates of the benefit–harm balance and decision making.

## Background

Roflumilast, a phosphodiesterase-4 inhibitor, has been approved, after a long regulatory process in the US and EU, for the prevention of exacerbations in patients with severe chronic obstructive pulmonary disease (COPD) and frequent exacerbations [1, 2]. Despite a carefully-conducted Cochrane systematic review based on high-quality randomized controlled trials (RCTs) [3], regulators, guideline developers, researchers and clinicians alike find it difficult to interpret these data [4–6]. Decisions or recommendations for or against drugs are commonly made under considerable uncertainty because it is unclear how relevant the beneficial (and statistically significant) effects are for (various) types of patients, who may or may not be vulnerable to particular side effects, or because long-term data and data from patients with comorbidities and co-medications are lacking [7–9].

Regulatory agencies, guideline developers, researchers and funding agencies commonly ask for more research when faced with such uncertainties. Often, such calls are not explicit with respect to defining what the uncertainty is about and how specific further research is likely to reduce it. Greenhalgh called the statement “more research is needed” the “most over-used and under-analyzed statement in the academic vocabulary” [10]. Others called for a 10-year moratorium on trials (“No More Cookbook Randomized Controlled Trials”) and a greater focus on the needs of practitioners, patients, payers, and policymakers in order to prioritize research [11]. The main sources of uncertainty, however, may be difficult to identify. Assessing how consequential specific additional research will be in order to augment the existing evidence can be hard, too.

Box 1 shows the research needs identified by the Cochrane review on roflumilast [3]. Nearly all of these needs can be addressed by RCTs. While each of the needs addresses an uncertainty about the evidence base, no prioritization is suggested.

Research prioritization is challenging because various stakeholders have their own perspectives and interests [12]. For example, the researchers themselves may ask for more research that fits their research agenda [13]. Patients and the general public, if asked, might request research that informs their decisions for or against medical interventions [14, 15]. Funding agencies have an interest in groundbreaking research and in wisely spending scarce resources on research with high relevance for patients’ quality of life, morbidity and mortality [16, 17]. Additional stakeholders, such as public and private funding agencies, industry, payers, and politicians, may bring yet another set of preferences for research priorities.

### Approaches to research prioritization

Research prioritization has gained much interest over the past 20 years. The goal of research prioritization is to rank-order research questions for specific stakeholders (e.g., patients or policymakers). In 2004, Fleurence and Torgerson provided a framework of approaches to research prioritization [18], distinguishing between five groups of approaches: burden of disease, subjective methods, impact on clinical variation, payback expectations, and value of information analyses (VOI). Box 2 provides a brief description of these five approaches. Currently, subjective approaches are probably most commonly used, but VOI has gained popularity among larger funding agencies such as the National Institute for Health and Clinical Excellence in the UK, the Agency for Healthcare Research and Quality (AHRQ), the Patient-Centered Outcomes Research Institute, and the National Institutes of Health [19–22].

The choice of approach to research prioritization partly depends on whether disease areas and/or risk factors are rank-ordered, or within disease areas, whether specific research questions about causal factors, diagnostic procedures, prognostic factors, and treatments are to be prioritized. For example, funding bodies, such as AHRQ, which sometimes take a “burden of disease” approach [23, 24] and specifically ask for research proposals on diseases (e.g., cardiovascular disease, cancer or respiratory disease) or risk factors (e.g., smoking or physical inactivity) with high burden for society [17], or the Bill and Melinda Gates foundation, which typically prioritizes research on diseases with high burden for developing countries such as malaria, tuberculosis, or HIV [25]. From a health systems perspective, it may be attractive to identify areas of clinical care where there is much practice variation and to prioritize research that has the potential to limit practice variation around best practices and get some payback, for example, by determining how much of which type of health care is minimally required to ensure good patient outcomes [26–28].

When research priorities within disease areas (e.g., COPD) need to be set, the selected research questions are often about the (comparative) effectiveness of specific interventions (e.g., different drug treatments) across populations and outcomes, including costs. Subjective methods, where the patients’ and clinicians’ perspective can be brought in, are commonly used [13, 15, 29, 30]. Studying the impact on clinical variation, payback expectations, and VOI are also approaches to define research priorities within disease areas. VOI is arguably the most versatile approach that can be used for various tasks of research prioritization [19, 31].

### The benefit–harm balance as an additional dimension to assess the potential impact of additional evidence

When setting research priorities at the level of specific interventions and comparisons, we propose that it may, at least occasionally, be useful to focus on the benefit–harm balance as the key parameter to decide on research priorities. Estimating the benefit–harm balance is a core activity of regulatory agencies and clinical guideline developers who must decide for or against preventive or therapeutic drug or non-drug treatments. Patients and clinicians, more or less explicitly, consider the benefit–harm balance before making decisions. As so often, where there is uncertainty about the benefit–harm balance of a certain treatment, we argue that research that has the potential to reduce uncertainty should be prioritized, similar to VOI methods. Priorities should be set for research that potentially changes the current estimate of the benefit–harm balance or makes it more precise, and as a consequence of an updated benefit–harm balance, impacts on (variability of) decision making. If additional research is unlikely to change the current estimate of the benefit–harm balance, it is unlikely that it will have an impact on practice.

To illustrate that, we focus on the quantitative assessment of the benefit–harm balance of roflumilast [32]. Such quantitative assessments may include cost, but costs are beyond the scope of this article. Several reviews have discussed quantitative approaches for benefit–harm assessment [33–35]. Conceptually, it is useful to distinguish between quantitative approaches that deal with single or very few benefit and harm outcomes and those that deal with multiple benefit and harm outcomes, and multiple categories thereof (e.g., mild, moderate, and severe COPD exacerbations) [34]. For example, balancing the reduction in moderate to severe COPD exacerbations versus increase in any gastrointestinal or psychiatric harm is often made using a comparison of the number-needed-to-treat and number-needed-to-harm. Often, as we and others have argued [34, 35], this oversimplifies the problem and one may want to use more sophisticated statistical approaches that consider multiple benefit and harm outcomes as well as a benefit–harm metric. Examples for those approaches include multi-criteria decision analysis, transparent uniform risk benefit overview, or the approaches developed by the National Cancer Institute (NCI) [36] and the PROTECT consortium (**P**harmacoepidemiological **R**esearch on **O**utcomes of **T**herapeutics by a **E**uropean **C**onsortium) [37].

**Fig. 1:**
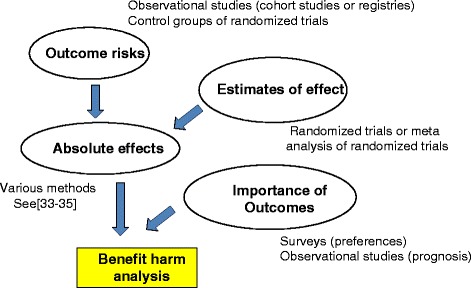
Key determinants of the benefit–harm balance of interventions. Figure adapted from Yu et al. [38]

Common to most approaches for quantitative benefit–harm assessment is that they are based on aggregated data, consider three key pieces of evidence as reported previously [38] (Fig. 1), and use statistical models to estimate the benefit–harm balance: treatment effects (typically available as relative treatment effect estimates from RCTs or meta-analyses), absolute outcome risks (available from observational studies or, if not, from control groups of RCTs), and importance of outcomes (i.e., which outcomes are more important than others, available from preference-eliciting surveys among patients or based on prognosis associated with the outcomes). Some approaches (e.g., benefit-less-risk analysis or the approach described by Boers et al. [39]) consider individual patient data, which allows for the consideration of the joint distribution of benefit and harm outcomes [39, 40]. Most quantitative benefit–harm assessments combine different data sources, each of which provide the best available estimates for treatment effects, outcome risks, and importance of outcomes (e.g., through patient preferences), respectively.

We know of no RCT that, on its own, provides enough data to estimate the benefit–harm balance. Often, a single trial, even if a large phase III trial, may provide precise estimates for the primary and some secondary outcomes. However, it is very unlikely that a single trial is powered enough for all outcomes including harms. This is even more true for baseline risk, where trials are often of limited value because the eligibility criteria (e.g., to make a trial as safe as possible) or selection of patients into trials may not give estimates of baseline risks that reflect real world patients. Further, some outcomes are just too rare so that baseline risks from a single trial are estimated imprecisely. Finally, trials rarely assess patient preferences. Although it would be welcome if preference elicitation surveys were embedded in trials, it rarely happens. Thus, RCTs may only under very exceptional circumstances provide the best available evidence for all three key pieces that are combined in quantitative benefit–harm assessments. Of course, the importance of careful and transparent selection of the most appropriate data for a quantitative benefit–harm assessment cannot be emphasized enough [34, 35, 37].

### Benefit–harm curves for illustrating the impact of additional research

**Fig. 2:**
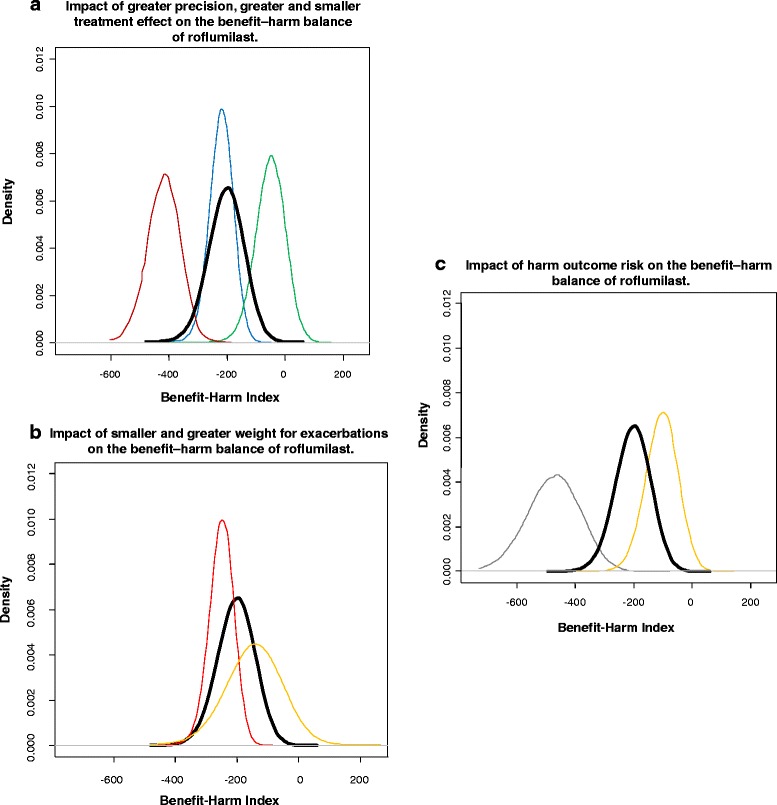
Benefit–harm curves for 100,000 estimates of the benefit-harm index (each curve) for roflumilast: IR, Incidence rate; IRR, Incidence rate ratio

We propose that quantitative benefit–harm assessment is valuable for setting research priorities as illustrated in Fig. 2a–c. We based these examples on a recent quantitative benefit–harm assessment of roflumilast that used the NCI approach (Box 3) [32]. One of the main analyses compared the expected outcomes of 10,000 male COPD patients below 65 years of age who received roflumilast over the course of 1 year with 10,000 male COPD patients below 65 years of age who did not receive roflumilast. We assumed that these patients had an intermediate (i.e., 30 %) risk of a moderate to severe exacerbation over the course of a year without roflumilast (i.e., the baseline risk), which corresponds to the approved indication for roflumilast.

The black (thick-lined) benefit–harm curve in Fig. 2a–c shows the distribution of 100,000 repetitions of the benefit–harm analysis for this scenario (10,000 male COPD patients <65 years with a 30 % 1-year risk of moderate to severe exacerbations). For each repetition, we calculated the index as the sum of benefit outcome events (i.e., prevented exacerbations) and harm outcome events (psychiatric, gastrointestinal, and neurological), based on a survival model and with weights as described in Box 3. The 100,000 repetitions take into consideration the statistical uncertainty of treatment effects on benefit and harm outcomes and of the outcome risks. A negative index means that the harms exceed the benefits. Almost all of the repetitions showed a negative index indicating that the probability that roflumilast is harmful for this scenario (male COPD patients <65 years with a 30 % 1-year risk of moderate to severe exacerbations) is very high, or, the probability of net benefit is close to 0 %.

We argue that research that has the potential to shift (change) or, if the benefit–harm curve overlaps with zero, can narrow the benefit–harm curves (i.e., make it more precise) should be prioritized. In addition, research that is likely to shift a benefit–harm curve in such fashion that the new curve can support different decisions (e.g., regulatory decisions or guideline recommendations) should also be prioritized. Since such decisions may refer to an entire population or subpopulations (e.g., COPD patients with severe disease and at high risk for exacerbations), additional studies may focus on subpopulations or use enrichment designs to have more statistical power for a particular subpopulation.

In Fig. 2a, the scenarios with evidence from additional RCTs are shown. If an additional RCTs comparing roflumilast with placebo became available, the most likely scenario is that it would not change the meta-analytic estimate of the exacerbation incidence rate ratio importantly, but narrow the curve because of a more precise (meta-analytic) effect estimate (shown by the blue curve). In another scenario, a very large additional RCT or a high-quality and large observational study could, if showing a much larger treatment effect, shift the meta-analytic treatment effect estimate towards a considerably larger value and thus shift the benefit–harm curve towards zero, increasing the probability that roflumilast provides net benefit (shown by the green curve). However, this scenario is unlikely given the stability (inertia) of the existing meta-analytic estimate and the substantial amount of evidence needed to cause such a major shift.

How about a RCT or an observational comparative effectiveness study where roflumilast is assessed as an add-on treatment to inhaled drug treatments? There have been some criticisms that the RCTs on roflumilast did not explicitly consider co-medications such as long-acting bronchodilators and inhaled corticosteroids, which raised concerns about the applicability of the trial results in real-world populations. In fact, the Food and Drug Administration (FDA) asked the manufacturer of roflumilast for a post-marketing commitment and “Conduct a controlled clinical trial to evaluate the efficacy of roflumilast as an add-on therapy to a long-acting beta agonist and inhaled corticosteroid fixed-dose combination therapy in the population of COPD patients for which roflumilast is indicated” [2]. To call for such a post-marketing commitment shows, similar to the research needs identified by the Cochrane review, a focus on research questions that can be addressed by RCTs.

However, it is unlikely that such a trial would show a larger treatment effect than existing trials that compared roflumilast against placebo on top of unclear co-treatments. Furthermore, the treatment effect can even be smaller, as is commonly the case in such head-to-head trials, and shrink the updated meta-analytic estimate. As a consequence the benefit–harm curve would shift to the left indicating an even more negative benefit–harm balance (red curve). In fact, the RCT, which the FDA asked the manufacturer of roflumilast to conduct for a post-marketing commitment, has been published just recently [41]. This RCT found a similar effect of roflumilast on exacerbations in severe COPD patients using fixed combinations of inhaled corticosteroids and long-acting beta-2 agonists as in previous studies (incidence rate ratio of 0.87 [95 % CI, 0.75–1.00]) [41]. The limited additional information this trial provided supports our argument against conducting additional RCTs when the benefit–harm balance is unlikely to change. It sometimes is not a sensible investment and, from an ethical point of view, the conduct of such a trial, which may cost 100 million US$ or more [42], may be questioned since it is unlikely that the newly estimated benefit–harm balance would lead to a different conclusion compared to the benefit–harm balance based on existing evidence.

Figure 2b shows the impact of knowing more about the importance patients assign to moderate-to-severe exacerbations. There is not much evidence about the importance of exacerbations from a patient’s perspective relative to other outcomes such as the harms caused by roflumilast. A preference-eliciting survey among COPD patients may show that patients are, on average, as concerned about some of the harms (e.g., incidence of depression or anxiety) as they are about exacerbations so that the relative weight used for the benefit–harm analysis should be larger. As a consequence, the benefit–harm curve would shift to the left (Fig. 2b, red curve), or the survey may show that patients assign more weight to moderate-to-severe exacerbations relative to the harm outcomes, which would shift the curve towards zero (yellow curve). The examples in Fig. 2b suggest that additional evidence about patient preferences may be valuable to inform the benefit–harm balance of roflumilast since the benefit–harm curve is likely to shift.

In the case of roflumilast, there is considerable evidence on harmful effects as the Cochrane review and FDA documents show. However, it is unclear at what (absolute) risks COPD patients are to experience the gastrointestinal, psychiatric, and neurological outcomes without roflumilast and how much roflumilast increases those risks. For the black curve in Fig. 2c, we assumed, based on the placebo groups of the roflumilast trials (for insomnia and anxiety) and an observational study (for depression), that the incident rates for depression, anxiety and insomnia are around 15 per 1,000 person years. There is some uncertainty about these incidence rates since risks in placebo groups often do not reflect risks observed in real-world populations because of the eligibility criteria and selection mechanisms in RCTs. Figure 2c illustrates that additional evidence, which is more valid and applicable for COPD patients in whom roflumilast is prescribed in real-world practice, may shift the benefit–harm curve considerably. If the incident rate of psychiatric outcomes is in fact higher than current estimates, which may well be the case, the benefit–harm curve would shift to the left (grey curve), meaning that the benefit–harm balance would become even less favorable. In this case, investment in such a cohort study with COPD patients may not be worthwhile since the conclusion that the benefit–harm balance is unfavorable would not change. If, however, there is an indication that current estimates of the incidence rates for depression, anxiety, and insomnia are overestimated, research in such an observational study may be justified because the benefit–harm curve would shift to the right indicating a more favorable benefit–harm balance for roflumilast than current estimates suggest (orange curve).

## Conclusion

In situations where additional research for specific interventions in specific populations needs to be prioritized, we propose that investigators may use benefit–harm assessment as a way to prioritize research. Research prioritization should not only focus on questions that can be addressed by new RCTs, but include specific research that has the potential to shift current estimates of the benefit–harm balance.

Previous research has identified many methodologies for quantitative benefit–harm assessment and discussed the challenges to performing such an assessment [34]. Therefore, these methods, among others to prioritize research, should be assessed for their usefulness in the process of determining research priorities. The example of roflumilast shows that the benefit–harm balance is sometimes more likely to change with additional specific evidence on patient preferences and outcome risks than with updated meta-analytic treatment effect estimates based on additional RCTs. Therefore, we propose that quantitative benefit–harm assessments have the potential to explore the impact of additional research and to identify research priorities.

## Box 1: Research needs identified by a Cochrane systematic review [3]

### Patient population

- Subgroup analysis in patients with/without chronic bronchitis and with/without history of exacerbations
- Effect of phosphodiesterase-4 (PDE4) inhibitors in patients with frequent exacerbations
- Use of PDE4 inhibitors in acute exacerbations

### Intervention (no research needs identified)

#### Comparator

- A direct comparison of PDE4 inhibitors and inhaled corticosteroid (ICS), when used as add-on therapies to either tiotropium or long-acting beta-2 agonists, or both
- A direct comparison of either tiotropium or long-acting beta-2 agonists, or both, as add-on therapies to PDE4 inhibitors (± ICS)

#### Outcomes

- Longer-duration studies to look at the effect of PDE4 inhibitors on forced expiratory volume in 1 second (FEV1) decline and mortality
- Effect of PDE4 inhibitors on healthcare utilization, including hospitalization (incidence and bed days)
- Effect of roflumilast on quality of life
- Better characterization of the weight loss seen with PDE4 inhibitors in COPD
- Better description of the nature of the effect on the exacerbations that do occur
- Cost-effectiveness of PDE4 inhibitors
- Ascertaining exercise tolerance data for roflumilast
- Determining if there is any benefit on cardiovascular outcomes with PDE4 inhibitors in COPD

#### Other

- Using the effects of PDE4 inhibitors to better understand the pathophysiology of COPD

## Box 2: Approaches for research prioritization (organized according to Fleurence and Torgerson [18])

### Burden of disease

A common approach to prioritize research is to use measures of disease burden such as mortality, disability-adjusted life years, cost of illness, or others. As a result, there is an enormous body of evidence on risk factors and (preventive) treatments for major cardiovascular diseases, major infectious diseases (e.g., HIV), major chronic lung diseases, and major cancers as opposed to scarce literature on risk factors and treatment effectiveness for rare(r) diseases.

### Subjective methods

Subjective methods employ expert knowledge and experiences to define areas of research that warrant higher priority than others. Subjective methods have been criticized because they are usually not transparent. However, more recently, a number of approaches have been developed that use knowledge and experience of experts in a more systematic and replicable way and in combination with systematic reviews.

### Impact on clinical variation

This approach assumes that variations in clinical practice result from uncertainty about the (comparative) effectiveness of interventions and disagreement among health care providers. Additional research is prioritized that has the potential to reduce uncertainty and, thereby, reduce variations in clinical practice. The Women’s Health Initiative trial is an example of how a randomized controlled trial (RCT) may change prescription rates (in this case, of post-menopausal hormone replacement therapy).

### Payback expectations

This approach foresees the impact of research on clinical variation but also takes into consideration the cost of additional research (e.g., a large-scale RCT). Payback refers to the money that is saved in the future as a result of the investment in research.

### Value of information

Value of information analysis estimates the impact of research on health outcomes and cost including the costs of conducting the research. Value of information analyses make the key determinants of uncertainty about the (comparative) effectiveness of interventions explicit and uses extensive modelling techniques to predict how additional research reduces uncertainty, and as a consequence, how the implementation of a new or an alternative intervention in practice and (parts of the) population will change health outcomes. This approach is highly flexible and allows prioritizing research in diverse contexts.

## Box 3: Summary of a recent quantitative benefit–harm analysis on roflumilast for patients with COPD [32]

We used the approach developed by Gail/NCI [36] estimating the probability that the benefits of an intervention exceed the harms for COPD patients at different risks for exacerbations. We considered the effects of roflumilast on exacerbations and harm outcomes, including gastrointestinal (acute pancreatitis, diarrhea, nausea, and weight loss), psychiatric (insomnia, anxiety, depression, and suicide), and neurological (headache and dizziness) symptoms or disorders based on high-quality RCTs that were included in the Cochrane review on roflumilast [2] and in the extensive evaluations of the US FDA [3]. Since harmful effects were expected from roflumilast, the trials paid particular attention to harm outcomes and reported remarkably precise estimates of treatment effects on harm outcomes and baseline risks, which was valuable for our benefit–harm analysis. Where trials did not provide valid or precise estimates of baseline risks we used data from the Centers for Disease Control and Prevention and from observational studies as reported previously [32]. We varied the outcome risk and severity of COPD exacerbations (moderate exacerbations defined as those requiring outpatient treatment, usually with systemic corticosteroids ± antibiotics, and severe exacerbations defined as those requiring hospital admission ± mechanical ventilation) as well as the weight of different benefit and harm outcomes. Thereby, we could determine the benefit–harm balance of roflumilast for various scenarios, clarifying considerably its potential indications.

We used moderate to severe exacerbations as the benefit outcome and included 10 gastrointestinal, psychiatric, and neurological events as harm outcomes. In the main analysis, we assigned a (relative) weight of 1.0 to completed suicide, 0.5 to incident moderate to severe exacerbations and acute pancreatitis, 0.25 to incident depression, anxiety and insomnia, and 0.05 to incident diarrhea, nausea, weight loss, headache and dizziness.

## Abbreviations

AHRQ: Agency for Healthcare Research and Quality
COPD: Chronic obstructive pulmonary disease
FDA: Food and Drug Administration
RCT: Randomized controlled trial
VOI: Value of information
